# Assessment of sleep quality and sleep disordered breathing among post-hospitalized patients with COVID-19

**DOI:** 10.3389/frsle.2023.1214036

**Published:** 2023-07-18

**Authors:** Paula L. Castellanos, Parthkumar Satashia, Mantavya Punj, Pablo R. Castillo, Brendon M. Colaco, Brynn K. Dredla, Emir Festic, Joseph Kaplan, Chad M. Ruoff, Leigh L. Speicher, Katherine L. Walsh, Natalia Werninck, Mingyuan Yin, Charles D. Burger, Vichaya Arunthari, Joseph Cheung

**Affiliations:** ^1^Department of Allergy, Pulmonary and Sleep Medicine, Mayo Clinic, Jacksonville, FL, United States; ^2^Department of Neurology, Mayo Clinic, Jacksonville, FL, United States; ^3^Department of Pulmonary and Sleep Medicine, Mayo Clinic, Scottsdale, AZ, United States; ^4^Department of General Internal Medicine, Mayo Clinic, Jacksonville, FL, United States

**Keywords:** COVID-19, SARS-CoV-2, OSA, long COVID, post-acute sequelae of COVID-19

## Abstract

**Background and objectives:**

We conducted a cross-sectional study to identify the presence of sleep disturbance and sleep disordered breathing in post-hospitalized patients with COVID-19 compared to a cohort of patients with no prior COVID-19 infection.

**Methods:**

Patients who were discharged from Mayo Clinic after hospitalization for COVID-19 and who had no existing diagnosis of sleep apnea or other sleep disorders were recruited for this study as cases. Patients who never had COVID-19 infection, nor any existing diagnosis of sleep apnea or other sleep disorders, were recruited from outpatient clinics as controls. Participants completed Pittsburgh Sleep Quality Index, Epworth Sleepiness Scale, and Fatigue Severity Scale questionnaires, as well as a home sleep apnea test.

**Results:**

Forty-seven COVID-19 cases and 46 controls completed questionnaires. Cases were significantly older, with a median age of 56.0 vs. controls (50.5) and were found to have slightly worse sleep quality, a higher degree of daytime sleepiness, and a slightly higher degree of fatigue. In terms of sleep disordered breathing, 39 cases and 40 controls completed the home sleep apnea test. Obstructive sleep apnea, defined by an AHI score of 5 or higher, was found in a remarkable 97.4% of cases vs. 72.5% in controls. Severity of OSA also trended higher in the case group. However, the difference in AHI was not significant after adjusting for age and BMI.

**Conclusion:**

Patients who were hospitalized with COVID-19 showed a very high prevalence of OSA. In addition, they had a slightly higher degree of sleep disturbance, daytime sleepiness, and fatigue when compared to controls. Our results suggest that sleep medicine assessment in patients who had COVID-19 requiring hospitalization is warranted.

## 1. Introduction

The COVID-19 pandemic has led to significant morbidity and mortality, especially in patients stricken with severe disease. Risks for developing severe disease and adverse outcomes include advanced age, male sex, morbid obesity, diabetes, and respiratory and cardiovascular diseases (Cariou et al., 2020; Booth et al., 2021). The burden of severe disease and adverse outcomes has decreased with the introduction of effective vaccines and therapeutics which, in turn, has led to a shift toward addressing the chronic sequelae of having the COVID-19 infection (Booth et al., 2021).

Multiple studies have examined the overlap between obstructive sleep apnea (OSA) and COVID-19. SARS-CoV-2 enters cells via angiotensin-converting enzyme 2 (ACE-2) receptors, which show increased expression in obese patients with OSA (Iannelli et al., 2020). COVID-19 has led to cardiac complications such as arrythmia, myocardial infarction, myocarditis, and acute heart failure, while OSA is a risk factor for similar cardiac pathology (Kohli et al., 2011; Bandyopadhyay et al., 2020). OSA-related hypoxia promotes a procoagulation state in patients; on the other hand, COVID-19–induced cytokine storm leads to increased clotting tendency (Hong et al., 2017; Hadid et al., 2021).

Longitudinal follow-up of prior severe acute respiratory syndrome (SARS) survivors has shown persistent burden of chronic fatigue at the 4-year follow-up mark (Lam et al., 2009). Fatigue is also a common symptom noted in patients who have recovered from acute COVID-19 (Wu et al., 2021). Fatigue itself can be secondary to numerous etiologies, including sleep-related disorders. OSA may also present with fatigue in addition to excessive daytime sleepiness and snoring.

Post-acute sequelae of COVID-19 (PASC, commonly known as Long COVID) has been proposed to have cardiopulmonary sequelae in a manner similar to that seen in OSA (Raman et al., 2022). These findings, along with the emergence of PASC, raises the question of whether COVID-19 itself increases the risk of developing OSA. One prior case study has reported a patient without any significant risk factors for OSA other than having COVID-19, who subsequently developed OSA as diagnosed on a home sleep apnea test (HSAT) (Nguyen and Mebust, 2021).

We conducted this cross-sectional study to assess for the burden of sleep disordered breathing (SDB) and sleep disturbances (SDs) in patients hospitalized with COVID-19 who did not have any prior documented history of SDB or sleep disorders.

## 2. Materials and methods

### 2.1. Subjects

This cross-sectional study recruited patients (cases) who were hospitalized at Mayo Clinic in Florida, Minnesota, or Arizona for COVID-19. All cases had been discharged at least 1 month prior to enrollment and were not on supplemental oxygen. Patients younger than 18 years, had a prior diagnosis of any sleep disorders including sleep-disordered breathing, chronic insomnia, sleep related movement disorders, central disorders of hypersomnolence, and/or pre-existing significant degree of cardio-pulmonary disease—congestive heart failure, active arrythmia, cardiomyopathy, pulmonary hypertension, or other causes of moderate to severe degree of obstructive or restrictive lung disease including COPD and Interstitial lung disease—were excluded from the study. Patients who presented for a primary care visit, never tested positive for COVID-19, and had no prior history of SDB or sleep disorders were recruited to participate in this study to serve as controls. An effort was made to group match controls to cases with regard to age, sex, and body mass index (BMI) to minimize bias and pretest probability between the 2 groups. This study was approved by the Mayo Clinic Institutional Review Board (IRB 20-012766).

### 2.2. Data collection

SDs were assessed using electronic questionnaires: the Pittsburgh Sleep Quality Index (PSQI), Epworth Sleepiness Scale (ESS), and Fatigue Severity Scale (FSS) administered via REDCap. The PSQI is a validated questionnaire which has been shown to have a sensitivity of 89.6% and specificity of 86.5% for detecting cases of a sleep disorder with a global PSQI score higher than 5 (Buysse et al., 1991, 2008). ESS is a validated questionnaire for detecting degree of sleepiness and scores of 10 or higher, indicating excessive daytime sleepiness (Johns, 1991, 2000). In comparison, FSS represents a validated questionnaire with scores of 4 or higher, indicating clinically significant fatigue (Valko et al., 2008).

SDB was evaluated using WatchPAT One (ZOLL Itamar), an FDA-approved, disposable home sleep testing device. Participants were asked to wear the WatchPAT One device for 1 night at home (Bar et al., 2003). The device uses an algorithm to assess for sleep and SDB by measuring peripheral arterial tonometry, heart rate, and oxygen saturation and provides other useful metrics such as sleep duration, sleep stages, body position, and snoring. WatchPAT One results were auto-scored, then followed with manual rescoring as needed. The device calculates apnea-hypopnea index (AHI), oxygen desaturation index (ODI), and time spent with SpO_2_ ≤ 88% (Jung et al., 2016). These indices are used to diagnose and define severity of SDB. The AHI represents the average number of hypopneas and apnea episodes per hour using AASM criteria with the 3% oxygen desaturation rule (Hudgel, 2016). The severity of OSA is defined as follows: AHI fewer than 5 events/h, no significant OSA; AHI 5–15 events/h, mild OSA; AHI 15–30 events/h, moderate OSA; and AHI more than 30 events/h, severe OSA.

### 2.3. Statistical analysis

Categorical variables were summarized as frequency (percentage) and continuous variables were reported as median (range) and mean (SD). Continuous and categorical variables were analyzed using the Wilcoxon rank sum test and Fisher exact test, respectively. Multivariable linear regression model was used to compare sleep and fatigue scores between cases and controls after controlling for baseline difference(s). All tests were 2-sided, with *P*-value < 0.05 considered statistically significant. The analysis was done using R version 3.6.2.

## 3. Results

### 3.1. Assessment of SD

Ninety-three patients (cases, *n* = 47; controls, *n* = 46) completed the electronic questionnaires. Cases were significantly older compared to controls (Table 1). Global PSQI score (median = 9 vs. 6, *P* < 0.001), ESS (median = 8 vs. 4, *P* = 0.002) and FSS (median = 3.3 vs. 2.5, *P* < 0.001) were all significantly higher for cases compared to controls before (Table 2) and after adjusting for age, sex, BMI, and AHI (AHI included only for patients who completed WatchPAT One test) (Tables 3–5).

**Table 1:** Demographic data.

	**Case (*N* = 47)**	**Control (*N* = 46)**	**Total (*N* = 93)**	***P-*value**
**Age**				0.045
Median (range)	56.0 (26.0–74.0)	50.5 (24.0–69.0)	51.0 (24.0–74.0)	
Mean (SD)	53.9 (13.1)	47.7 (14.3)	50.8 (14.0)	
**Sex**				0.20
Female, No. (%)	25 (53.2)	31 (67.4)	56 (60.2)	
Male, No. (%)	22 (46.8)	15 (32.6)	37 (39.8)	
**BMI**				0.74
Median (range)	31.1 (22.3–59.9)	31.1 (20.1–47.8)	31.1 (20.1–59.9)	
Mean (SD)	32.2 (6.8)	31.3 (6.9)	31.7 (6.9)	
**Hypertension**				0.083
No, No. (%)	26 (55.3)	34 (73.9)	60 (64.5)	
Yes, No. (%)	21 (44.7)	12 (26.1)	33 (35.5)	
**Diabetes mellitus**				0.55
No, No. (%)	39 (83.0)	41 (89.1)	80 (86.0)	
Yes, No. (%)	8 (17.0)	5 (10.9)	13 (14.0)	

BMI, Body mass index; SD, Standard deviation.

**Table 2:** Questionnaire assessment.

	**Case (*N* = 47)**	**Control (*N* = 46)**	**Total (*N* = 93)**	***P-*value**
**PSQI**				< 0.001
Median (range)	9.0 (2.0–18.0)	6.0 (1.0–21.0)	7.0 (1.0–21.0)	
Mean (SD)	9.3 (4.2)	6.5 (4.2)	7.9 (4.4)	
**ESS**				0.002
Median (range)	8.0 (1.0–18.0)	4.0 (0.0–19.0)	5.0 (0.0–19.0)	
Mean (SD)	8.0 (4.4)	5.4 (4.1)	6.7 (4.4)	
**FSS**				< 0.001
Median (range)	3.3 (1.0–7.0)	2.2 (1.0–6.2)	2.7 (1.0–7.0)	
Mean (SD)	3.6 (1.8)	2.5 (1.2)	3.0 (1.6)	

PSQI, Pittsburgh sleep quality index; ESS, Epworth sleepiness scale; FSS, Fatigue severity scale; SD, Standard deviation.

**Table 3:** Sleep quality (PSQI) in COVID-19 cases vs. controls (adjusted).

**Term**	**Beta (95% CI)**	***P-*value**
**Without AHI (*****N*** = **93)**		
Age (per 1-yr increase)	−0.06 (−0.12 to 0)	0.063
Male vs. female	−2.91 (−4.65 to −1.18)	< 0.01
BMI (per 1-point increase)	−0.02 (−0.14 to 0.1)	0.778
Case vs. control	3.6 (1.91 to 5.29)	< 0.01
**With AHI (*****N*** = **79)**		
Age (per 1-yr increase)	−0.01 (−0.07 to 0.06)	0.878
Male vs. female	−1.92 (−3.84 to 0)	0.054
BMI (per 1-point increase)	0.04 (−0.09 to 0.16)	0.584
AHI (per 1-point increase)	−0.06 (−0.13 to 0.02)	0.147
Case vs. control	3.33 (1.49 to 5.17)	< 0.01

BMI, Body mass index; AHI, Apnea-hypopnea index.

**Table 4:** Daytime sleepiness (ESS) in COVID-19 cases vs. controls (adjusted).

**Term**	**Beta (95% CI)**	***P-*value**
**Without AHI (*****N*** = **93)**		
Age (per 1-yr increase)	−0.05 (−0.11 to 0.01)	0.084
Male vs. female	−2.46 (−4.22 to −0.71)	< 0.01
BMI (per 1-point increase)	0.09 (−0.03 to 0.22)	0.137
Case vs. control	3.25 (1.54 to 4.95)	< 0.01
**With AHI (*****N*** = **79)**		
Age (per 1-yr increase)	−0.05 (−0.12 to 0.03)	0.21
Male vs. female	−3.08 (−5.08 to −1.08)	< 0.01
BMI (per 1-point increase)	0.08 (−0.05 to 0.21)	0.23
AHI (per 1-point increase)	−0.01 (−0.09 to 0.07)	0.85
Case vs. control	3.6 (1.68 to 5.51)	< 0.01

BMI, Body mass index; AHI, Apnea-hypopnea index.

**Table 5:** Fatigue (FSS) in COVID-19 cases vs. controls (adjusted).

**Term**	**Beta (95% CI)**	***P-*value**
**Without AHI (*****N*** = **93)**		
Age (per 1-yr increase)	−0.02 (−0.04 to 0)	0.097
Male vs. female	−1.07 (−1.71 to −0.44)	0.001
BMI (per 1-point increase)	−0.04 (−0.08 to 0.01)	0.109
Case vs. control	1.47 (0.85 to 2.09)	< 0.001
**With AHI (*****N*** = **79)**		
Age (per 1-yr increase)	−0.01 (−0.03 to 0.02)	0.45
Male vs. female	−0.95 (−1.65 to −0.24)	0.01
BMI (per 1-point increase)	−0.03 (−0.08 to 0.01)	0.19
AHI (per 1-point increase)	−0.01 (−0.04 to 0.01)	0.35
Case vs. control	1.6 (0.92 to 2.27)	< 0.01

BMI, Body mass index; AHI, Apnea-hypopnea index.

### 3.2. Assessment of SDB

Seventy-nine patients (cases, *n* = 39; controls, *n* = 40) completed the WatchPAT One test. Cases were significantly older compared to controls (median = 57 vs. 51 years, *P* = 0.019). Median duration of hospitalization for cases was 6 days (range, 3–15). Median duration between initial infection and completion of home sleep apnea testing was 140 days (range, 52–487). Among cases, 7 of 39 received non-invasive ventilation and only 1 required invasive ventilation during their hospitalization. Cases were found to be significantly more likely to have OSA (97 vs. 73%, *P* = 0.003), unadjusted; we are unable to adjust by age given low number of patients (total of 12) without OSA. Cases had a higher AHI (median = 15.6/h vs. 9.6/h, *P* = 0.017), ODI (median = 6.8 vs. 3.0, *P* = 0.009), and spent more time with SpO_2_ ≤ 88% (median = 0.2 vs. 0 min, *P* = 0.013) compared to controls, respectively (Table 6). However, when analyzing the difference in AHI as a continuous variable (OSA severity) between cases and controls, after adjusting for age and BMI, AHI was no longer statistically significant (Table 7). Central sleep apnea was not observed to any significant degree among cases or controls.

**Table 6:** Sleep disordered breathing parameters (unadjusted).

	**Case (*N* = 39)**	**Control (*N* = 40)**	**Total (*N* = 79)**	***P-*value**
**AHI**				0.017
Median (range)	15.6 (1.8–58.9)	9.6 (1.4–42.6)	10.9 (1.4–58.9)	
Q1, Q3	8.1, 25.4	4.0, 16.1	6.7, 21.4	
**AHI category**				0.003
< 5, No. (%)	1 (2.6)	11 (27.5)	12 (15.2)	
≥5, No. (%)	38 (97.4)	29 (72.5)	67 (84.8)	
**OSA severity**				0.013
No, No. (%)	1 (2.6)	11 (27.5)	12 (15.2)	
Mild, No. (%)	18 (46.2)	17 (42.5)	35 (44.3)	
Moderate, No. (%)	13 (33.3)	8 (20.0)	21 (26.6)	
Severe, No. (%)	7 (17.9)	4 (10.0)	11 (13.9)	
**TST, min**				0.11
Median (range)	402.0 (240.0–514.0)	384.5 (240.0–464.0)	387.0 (240.0–514.0)	
Q1, Q3	340.5, 461.5	323.2, 423.2	336.5, 432.5	
**RDI**				0.080
Median (range)	17.5 (2.0–58.9)	12.1 (4.7–43.4)	14.5 (2.0–58.9)	
Q1, Q3	10.6, 27.1	8.9, 20.4	9.6, 25.4	
**ODI**				0.009
Median (range)	6.8 (0.5–51.3)	3.0 (0.2–21.3)	4.1 (0.2–51.3)	
Q1, Q3	2.4, 12.8	1.1, 5.5	1.6, 9.1	
**Central apnea index**				0.21
Median (range)	0.6 (0.0–9.7)	0.4 (0.0–3.4)	0.5 (0.0–9.7)	
Q1, Q3	0.1, 1.0	0.0, 0.9	0.0, 1.0	
**Mean Sp** **O**_2_				0.12
Median (range)	94.0 (89.0–97.0)	94.0 (65.0–96.0)	94.0 (65.0–97.0)	
Q1, Q3	93.0, 94.5	93.0, 95.0	93.0, 95.0	
**Nadir Sp** **O**_2_				0.27
Median (range)	85.0 (51.0–92.0)	86.0 (73.0–94.0)	86.0 (51.0–94.0)	
Q1, Q3	82.0, 89.0	82.8, 90.2	82.5, 89.5	
**Time SpO**_2_ ≤ **88%**				0.013
Median (range)	0.2 (0.0–111.6)	0.0 (0.0–5.4)	0.1 (0.0–111.6)	
Q1, Q3	0.0, 3.1	0.0, 0.3	0.0, 0.9	
**Mean HR**				0.24
Median (range)	69.0 (52.0–98.0)	65.5 (45.0–90.0)	67.0 (45.0–98.0)	
Q1, Q3	62.0, 73.0	60.0, 73.0	62.0, 73.0	

AHI, Apnea-hypopnea index; OSA, Obstructive Sleep Apnea; RDI, Respiratory disturbance index; TST, Total steep time determined by WatchPAT device; ODI, oxygen desaturation index; SpO_2_, Oxygen saturation.

**Table 7:** Adjusted sleep disordered breathing parameters in COVID-19 vs. controls.

**Term**	**Beta (95% CI)**	***P-*value**
**Model predicting AHI**		
Age (per 1-yr increase)	0.36 (0.17 to 0.55)	< 0.01
Male vs. female	7.15 (1.53 to 12.78)	0.015
BMI (per 1-point increase)	0.2 (−0.17 to 0.57)	0.297
Case vs. control	2.86 (−2.64 to 8.36)	0.312
**Model predicting ODI**		
Age (per 1-yr increase)	0.23 (0.1 to 0.36)	< 0.01
Male vs. female	5.23 (1.31 to 9.16)	0.011
BMI (per 1-point increase)	0.14 (−0.13 to 0.4)	0.312
Case vs. control	3.14 (−0.7 to 6.98)	0.113
**Model predicting time of SpO**_2_<**88%**		
Age (per 1-yr increase)	0.22 (0.01 to 0.44)	0.045
Male vs. female	5.86 (−0.47 to 12.19)	0.074
BMI (per 1-point increase)	0.16 (−0.26 to 0.58)	0.451
Case vs. control	3.55 (−2.64 to 9.75)	0.264

BMI, Body mass index; AHI, Apnea-hypopnea index; ODI, Oxygen desaturation index; SpO_2_, Oxygen saturation.

## 4. Discussion

This cross-sectional cohort study compared the burden of SDs and SDB in patients (cases) discharged after hospitalization for COVID-19 to outpatients controls presenting to a primary care appointment without prior documented COVID-19 infection. It is important to note that in both cases and controls participating in this study, none of the participants had a prior diagnosis of a sleep disorder or SDB.

We assessed for SDs and daytime sleepiness in our 2 cohorts using the PSQI and ESS questionnaires. We found that cases had a global PSQI median score of 9 for cases vs. 6 in controls, suggesting poorer sleep quality and increased SD in cases compared with controls. These findings are consistent with existing literature that SD and insomnia have been observed in patients after hospitalization and critical illness (Altman et al., 2017; Ramani et al., 2021). In particular, in one study of post-ICU COVID-19 patients, 22% of patients reported a new-onset, moderate to severe degree of insomnia (Ramani et al., 2021). One consideration for the higher level of SD in cases is PASC (Chen et al., 2022) PASC involves a constellation of symptoms which persist for longer than 3 months after COVID-19 and continue for at least 2 months without any alternative etiology (Davis et al., 2021). The symptoms may include chronic fatigue, pain, cognitive dysfunction, and mood-related disturbances (e.g., anxiety and depression) (Davis et al., 2021; Chen et al., 2022). Previous studies evaluating longitudinal outcomes in COVID-19 hospital survivors have noted that a majority of COVID-19 survivors had functional and physical recovery; however, chronic sequelae (e.g., dyspnea, lung diffusion impairment, radiographic pulmonary abnormalities, and fatigue) and SDs persisted, even at the 12-month mark (Huang et al., 2021; Wu et al., 2021).

A study examining prevalence of symptoms across multiple organ systems at 7 months post-COVID-19 infection found a high prevalence of SDs (Davis et al., 2021). Prior estimates of the global prevalence of Long COVID were estimated at 43%, but were greater for patients hospitalized for COVID compared to those who were not (54 vs. 34%) (Adab et al., 2022). Recently, the COVID-19 Task Force of the American Academy of Sleep Medicine suggested that clinicians query post-COVID patients regarding their sleep, given that SDs are a common feature of PASC (Khosla et al., 2023).

We found that cases had an ESS median score of 8 vs. 4 in controls, suggesting a higher degree of daytime sleepiness among cases. This is consistent with data from other studies and is indicative of SDs, and likely daytime sleepiness, representing a unique sequelae for post-hospitalized COVID-19 patients. Importantly, our results of the PSQI and ESS scores remained significant after adjusting SDB (AHI). Pathologic mechanisms behind these findings could be related to post-acute neurologic sequelae due to SARS-CoV-2 relating to possible vascular injury, neurotransmitter dysfunction, and effects relating to neuroinflammation (Boldrini et al., 2021; Alzueta et al., 2022). Another longitudinal study noted that alterations in the brain interstitial fluid metabolic waste clearance could be a mechanism via poor sleep quality after COVID-19 (Del Brutto et al., 2022). The structural changes could represent possible mechanisms for daytime sleepiness as well.

In comparison, FSS represents a validated questionnaire, with scores of 4 or greater indicating fatigue; in this study, cases had a median score of 3.3 vs. controls (median, 2.2). Although our results did not indicate a larger clinical difference for fatigue between cases and controls, other studies have noted a larger burden. One single-center study evaluating post-discharge symptoms noted that sleep disorders and fatigue were among the more common reported symptoms (Garrigues et al., 2020). Another consideration for higher PSQI, ESS, and FSS among cases could be attributed the hospitalization itself. A recent review provides a framework for consideration of psychiatric sequelae in patients after COVID-19 critical illness (Sankar et al., 2022). This includes patients experiencing depression, anxiety, and post-traumatic stress disorder, all of which could contribute to a higher burden of fatigue, daytime sleepiness, and SDs.

Large-scale analysis of electronic medical data found patients with OSA had an 8-fold higher risk for COVID-19 infection (Maas et al., 2021). As such, studies have examined a potential association between OSA and COVID-19 (Bhatraju et al., 2020; Cade et al., 2020; Richardson et al., 2020). One such study identified treated OSA as an independent risk factor associated with an increased risk of death on the seventh day of COVID-19–related hospitalization in diabetic patients (Cariou et al., 2020). Patients with COVID-19 have also been shown to have manifestations of pulmonary fibrosis, which itself has been shown to be related to development of OSA (Lancaster et al., 2009; Ye et al., 2020). Remarkably, our study found a very high prevalence of OSA at 97.4% (38 of 39) in our COVID-19 cases, and was significantly more so than controls at 72.5% (29 of 40). When we analyzed the difference in AHI as a continuous variable between cases and controls after adjusting for age and BMI in our regression model, however, AHI was no longer statistically significant. We acknowledge that our small sample size may have precluded detection of a difference since other studies have demonstrated an increased prevalence of SDB in COVID-19 patients. We also attribute some of the lack of statistical significance to a high percentage of previously undiagnosed OSA in our controls due to this cohort being older and with a higher BMI, which are comorbidities/predisposing factors for OSA, than the normal population, but selected into the study in order to best match the demographics of our cases.

Age and obesity are risk factors associated with increased OSA prevalence (Young et al., 1993). The angiotensin-converting enzyme 2 (ACE2) receptor, which belongs to the renin-angiotensin-aldosterone system, has been implicated in the ability of the SARS-CoV-2 virus to enter human cells (Yan et al., 2020). One consideration is obesity itself having increased ACE2 and dysregulation of the renin-angiotensin-aldosterone system (RAAS) and, in turn, being associated with increased severity of COVID-19 (Iannelli et al., 2020). Untreated OSA has been implicated with increased expression of angiotensin-converting enzyme (Gruber et al., 2006). A recent study examined risk of OSA in patients with acute respiratory distress syndrome (ARDS) due to COVID-19 and found that 49 of 67 had moderate to severe OSA on polysomnography 4–6 weeks after discharge (Goyal et al., 2022). There is also the consideration that patients who had a serious course of COVID-19 were referred for further evaluation for OSA as part of their post-hospital follow-up, which may have led to more severe cases not meeting our inclusion criteria. This is in light of a retrospective analysis in 2021, which showed that having OSA was associated with an elevated risk of progression to severe COVID-19, including requiring ICU admission (Hariyanto and Kurniawan, 2021). Lastly, there is also the possibility that there is no link between SDB and a history of having COVID-19, as discussed in a previous retrospective study which did not find OSA to be an independent risk factor for worse COVID-19 outcomes for hospitalized patients (Mashaqi et al., 2021). Similarly, another study found no relationship between having untreated OSA, in the setting of resource limitations in a developing country, and the risk of having a more severe course of COVID-19 disease (Del Brutto et al., 2021).

Our study has several limitations. First, we recognize that our sample size is small (*n* = 93), particularly in the assessment of SDB portion of the study (*n* = 79). Second, use of home sleep apnea tests to evaluate for SDB instead of using the gold standard attended overnight polysomnography, which has higher diagnostic accuracy. We attempted to closely match for comorbidities and demographic factors between cases and controls; however, cases were slightly older than controls. In addition, cases were hospitalized for a median of 6 days, whereas controls were recruited from the outpatient population. Additionally, milder, non-hospitalized cases of COVID-19 were not included in the study. Lastly, although the controls did not have a documented history of COVID-19, confirmatory serologic testing was not done due to resource constraints.

Our study suggests that COVID-19 patients post-hospitalization have a higher burden of SDs, poor sleep quality, and daytime sleepiness. They also had a higher prevalence of SDB; however, our data did not indicate a significant difference in the apnea-hypopnea index after considering age and BMI. These findings suggest that COVID-19 patients warrant a multidisciplinary approach following hospitalization, including proactive screening for SDB and SDs.

## Data availability statement

The original contributions presented in the study are included in the article/supplementary material, further inquiries can be directed to the corresponding author.

## Ethics statement

The studies involving human participants were reviewed and approved by Mayo Clinic IRB. The patients/participants provided their written informed consent to participate in this study.

## Author contributions

JC, PLC, and PS take responsibility for (are the guarantors of) the content of the manuscript and including the data and analysis. PLC and PS collected the data. All authors played a role in the content and writing of the manuscript. All authors contributed to the article and approved the submitted version.
